# Interpretable prognostic modeling of endometrial cancer

**DOI:** 10.1038/s41598-022-26134-w

**Published:** 2022-12-13

**Authors:** Bulat Zagidullin, Annukka Pasanen, Mikko Loukovaara, Ralf Bützow, Jing Tang

**Affiliations:** 1 Research Program in Systems Oncology, Faculty of Medicine, University of Helsinki, 00290 Helsinki, Finland; 2 Institute for Molecular Medicine Finland (FIMM), HiLIFE, University of Helsinki, 00290 Helsinki, Finland; 3 Department of Pathology, University of Helsinki and Helsinki University Hospital, 00290 Helsinki, Finland; 4 Department of Obstetrics and Gynecology, Helsinki University Hospital and University of Helsinki, 00290 Helsinki, Finland; 5 Research Program in Applied Tumor Genomics, Faculty of Medicine, University of Helsinki, 00290 Helsinki, Finland; 6 Department of Biochemistry and Developmental Biology, Faculty of Medicine, University of Helsinki, 00290 Helsinki, Finland

**Keywords:** Translational research, Endometrial cancer, Molecular medicine, Prognostic markers

## Abstract

Endometrial carcinoma (EC) is one of the most common gynecological cancers in the world. In this work we apply Cox proportional hazards (CPH) and optimal survival tree (OST) algorithms to the retrospective prognostic modeling of disease-specific survival in 842 EC patients. We demonstrate that linear CPH models are preferred for the EC risk assessment based on clinical features alone, while interpretable, non-linear OST models are favored when patient profiles can be supplemented with additional biomarker data. We show how visually interpretable tree models can help generate and explore novel research hypotheses by studying the OST decision path structure, in which L1 cell adhesion molecule expression and estrogen receptor status are correctly indicated as important risk factors in the p53 abnormal EC subgroup. To aid further clinical adoption of advanced machine learning techniques, we stress the importance of quantifying model discrimination and calibration performance in the development of explainable clinical prediction models.

## Introduction

Endometrial carcinoma (EC) is the most common gynecologic malignancy in the OECD member states. In 2020, 417,000 new cases and 97,370 deaths have been attributed to the EC worldwide, which is a 10% increase in incidence and an 8% increase in mortality since 2018. Both metrics vary considerably geographically and across patients’ socioeconomic strata^1,2^. In the UK, the expected 5-year survival is 77%, with 85% for stage I disease and 25% for stage IV^3^. EC treatment options depend on tumor staging and histological findings, which are prone to misdiagnosis^4^. Addition of molecular profiling information to histological features has been shown to improve patient stratification and subsequent selection of adjuvant therapies^5–9^. To further improve the EC risk assessment, it is important to develop transparent computational models that utilize both clinical and molecular patient profiles.

Two commonly used statistical methods in the survival analysis of EC patients are the Kaplan–Meier method and the Cox proportional hazards (CPH) regression. The Kaplan–Meier method is used to approximate cumulative survival probability (survival function) from lifetime and censored data^10^. It is well-suited to summarize survival functions from full cohorts, and it allows for their visual analysis. The CPH regression is the most popular model for the analysis of survival data when multiple variables are available^11^. Its utility is limited due to the CPH assumptions, such as the linearity and additivity of predictor variables, as well as the methodological difficulties related to variable selection. Machine learning (ML), such as deep learning and ensemble models, improve on these shortcomings. They have been shown to perform particularly well with high-dimensional datasets, such as -omics readouts, electronic health records, and high content imaging^12,13^. Deep learning and ensemble ML models have also been applied to prognostic prediction modeling of patient outcomes in the EC^14–17^. However, these ML models still see limited use in the clinical practice^18^. Their poor adoption may be attributed to the black-box nature that complicates model interpretability, a high risk of bias, and the need for larger training datasets to achieve similar performance, as compared to linear Cox regression^19^.

Tree-based ML methods have been used to account for non-linear effects and variable interactions in survival analysis^20^. Tree-based ML methods are interpretable by design as every prediction made by a trained model can be associated with a corresponding decision path, and the hierarchical structure of the model as a whole can be easily visualized. Further, they can take into account factors that may act differently in patient subgroups, unlike linear models that favor global factors with uniform effects across entire patient cohorts^21^. There are several variants of decision trees that can be used to estimate patient risks, such as the CART model proposed by Breiman et al. or the conditional inference tree model by Hothorn et al.^22,23^. While decision trees can be ensembled leading to better performance than single trees, like in the random survival forest algorithm by Ishwaran et al., this makes them considerably less interpretable^24,25^. In light of recent research advances aimed at improving decision tree algorithms through better splitting and pruning criteria, single decision tree models are a good alternative to the CPH regression in the development of explainable clinical prediction models^26,27^.

In this retrospective study we explore a cohort of 842 EC patients with 43 clinicopathological and molecular features collected at the Helsinki University Hospital between 2007 and 2012. We report two interpretable models that predict disease-specific survival: a multivariable CPH regression and a visually interpretable optimal survival tree (OST)^27^. Both are built on two sets of variables: a clinical set and an extended set, which enriches the former with biomarker data, namely CD171 (L1CAM, L1 cell adhesion molecule expression), estrogen receptor (ER) status, peritoneal washing and tumor size. We use Harrell’s time-independent concordance index (C-index) and time-dependent integrated Brier score (IBS) to compare model performance^28^. These two measures report related, but distinct performance metrics, as C-index quantifies discrimination, or how well a model separates low-risk from high-risk patients, while IBS also quantifies calibration, which is the extent of an agreement between observed outcomes and model predictions^29^. In this work we show that to select an optimal EC prognostic model, a discrimination measure should be supplemented with a calibration measure, such as IBS^30–33^. We find that the CPH models trained on the clinical variables have a higher C-index than the OST models, whereas the IBS scores of both model types are comparable. Extending clinical data with biomarker information improves the discrimination and calibration performance in both model types, with a larger improvement and the overall best C-index and IBS scores seen in the OST models. Finally, we suggest that the Cox proportional hazards regression should be used in the EC risk assessment based on clinical data only, while optimal survival trees are preferred when biomarker information is available.

## Materials and methods

### Study cohort

This retrospective analysis is based on a cohort of 842 patients with unselected EC that underwent surgical treatment between 2007 and 2012 at the Helsinki University Hospital. The follow-up time ranges from 1 to 136 months with a median of 82 months. In total, 591 (70.2%) patients survived until the end of the study, 148 (17.6%) died from the EC, 103 (12.2%) died from other causes. The endpoint of interest is disease-specific survival. Based on tumor molecular profiles derived through The Cancer Genome Atlas project, 604 (71.7%) patients were assigned to one of four ProMisE classes, for the remaining 238 (28.2%) patients the ProMisE categories were not assigned experimentally^5,6^. Four categories are: (a) mismatch repair deficient (MMRd), (b) no specific molecular profile (NSMP), (c) p53 abnormal and (d) polymerase-ε hypermutated (POLE). Among 604 patients that have ProMisE classes assigned to them, 74 died due to other causes and 30 belong to the POLE subgroup, where no one died from the EC. Each patient is described with a feature vector consisting of 43 variables, out of which 33 are categorical and 10 are numeric. Please refer to the Supplementary Materials—Extended variable information for a more detailed variable description.

### Data preprocessing

All numeric variables, except for age and BMI, are winsorized at the 99% level to limit the effect of extreme values using the quantile function derived via the inverse of an empirical distribution function^34^. Variables with more than five categories, such as FIGO stage, or those with unbalanced class proportions, such as adjuvant therapy status, are simplified by combining subcategories together.

We impute missing values to prevent the exclusion of observed data^35^. Missing values are imputed using the multivariate imputation by chained equations method, where numerical and binary variables are predicted with random forest models consisting of 100 decision trees, unordered categorical data with more than two levels are imputed with the polytomous regression, and ordered categorical variables with more than two levels are imputed with the proportional odds model^36^. Variables are imputed in the order of low to high proportion of missingness. R mice package version 3.14.7 is used to generate 120 imputed datasets, which are subsequently merged by taking mean values for the numeric variables and mode values for categorical variables^37^. The response variable is kept throughout the imputation^38^.

Finally, to select variables for the CPH regression models we compare the distributions of numerical and binary categorical variables, stratified by the response. We apply the Pearson correlation coefficient to identify collinear numerical variables, and Goodman and Kruskal’s lambda to identify associated categorical variables. Our primary goal is to optimize the CPH regression performance. Therefore, simplification of categorical data and variable selection in the subsequent steps are iterated several times. We use the analysis of deviance test to compare nested CPH models, while the Akaike information criterion is preferred for the comparison of non-nested models.

**Figure 1:**
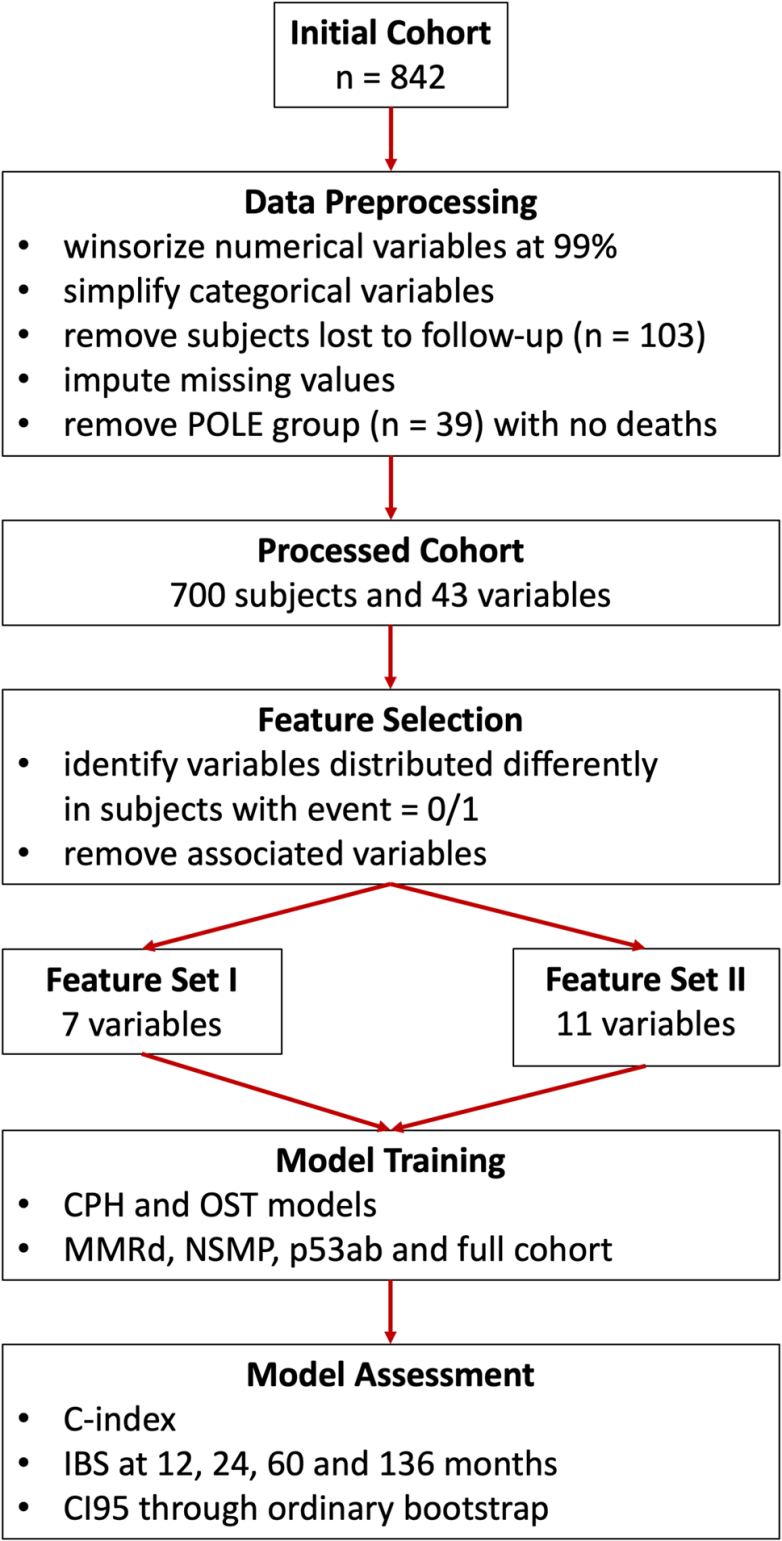
Experimental pipeline. POLE stands for polymerase-ε hypermutated ProMisE class, MMRd – mismatch repair deficient, NSMP – no specific molecular profile, p53ab – p53 aberrant, where ProMisE is Proactive Molecular Risk Classifier for Endometrial Cancer. CPH is Cox proportional hazards regression, OST – optimal survival tree, IBS – integrated Brier score and CI95 is 95% confidence interval.

The complete experimental pipeline is shown in Fig. 1.

### Statistical modeling

We train two types of interpretable models to predict individual survival probabilities for the full patient cohorts, and the subcohorts stratified by the ProMisE classes. We assess model performance using C-index and IBS. We estimate 95% confidence intervals for the performance metrics by 1000 repetitions of the ordinary bootstrap with replacement. We also report the performance of seven additional survival analysis models using the C-index metric in the Supplementary Materials—Additional ML models section. We follow the Transparent Reporting of a multivariable prediction model for Individual Prognosis Or Diagnosis (TRIPOD) Initiative hoping to decrease reporting bias and enable model interoperability^39^.

### Cox proportional hazards model

Survival CPH regression is defined as a product of a non-parametric hazard function *λ(t)* and the *e*^*Xβ*^ term, where *t* is time, *X* is a vector of variables describing a patient, and *β* is a vector of the model's coefficients. The *λ(t)* part of the CPH model is identical for all patients at a time *t*. It is referred to as a hazard function of a standard patient, which is a patient with *Xβ* = 0. The second term is patient-specific, and it is used to calculate a hazard ratio without knowing the hazard function *λ(t)*, where the hazard ratio is the risk of death in relation to a control^40^. We use the Breslow method, as implemented in R riskRegression package version 2022.03.22, to specify the hazard function, which is required for estimating individual survival probabilities^41^. We use Schoenfeld residuals to test for the proportional hazards assumption, as implemented in R survival package version 3.3.1. We estimate CPH model parameters by maximizing the partial log-likelihood.

### Optimal survival tree model

We use the optimal survival tree (OST) method to develop interpretable decision tree models for estimating patient survival probabilities. The OST algorithm creates multiple candidate decision trees and optimizes their variable splitting thresholds one variable at a time using coordinate descent^42^. The main idea is to use previously optimized parameters in subsequent splitting criteria updates, ultimately outputting a single decision tree that can be visually examined. The OST loss function compares how close the predicted *e*^*Xβ*^ terms for each patient are to the cumulative survival probabilities, obtained by the Nelson-Aalen estimator^27^. We prioritize model robustness in the training process by: (a) limiting the tree size, since too deep or too wide trees obfuscate the model interpretability, (b) increasing the number of random restarts to use in the local search algorithm, and (c) controlling the minimum number of points that must be present in every leaf node of the fitted trees. The complexity parameter that determines the tradeoff between the accuracy and model complexity is tuned automatically by assessing the out-of-sample performance. The patient cohorts for the OST model training are split, such that the complete cases are used for model fitting, and the imputed subsets are used for validation. The final validated models are then retrained on the combined (complete case and imputed) patient cohorts. We fit the OST models, as implemented in R iai package version 1.7.0, using the log-likelihood criterion^43^.

### Model performance metrics

C-index reports model discrimination performance, i.e. the model’s ability to predict correct rankings of the survival times. C-index is defined as a ratio of concordant pairs of subjects to the total number of comparable pairs. A pair is concordant when a subject with shorter survival time is estimated to have a higher risk than the one with longer survival time. A pair is comparable if (a) it is possible to determine which subject experienced the event first or (b) a subject with a shorter survival time experienced an event, while the other one is censored and is not lost to follow-up yet. C-index ranges between 0 and 1, where higher values are better.

IBS reports both model discrimination and calibration, i.e. the extent of an agreement between observed outcomes and model predictions^44^. Brier score is defined as a mean squared difference between event indicators and predicted survival probabilities at a time *t*^28^. By summing Brier scores over a time interval we obtain the integrated Brier score (IBS), which is then adjusted for patients lost to follow-up using the inverse probability censoring weighting method^45^. We use R pec package version 2022.05.04 to compute IBS at 12, 24, 60, and 136 months based on the predicted individual survival probabilities of patients. IBS ranges between 0 and 1, where lower values are better.

### Computational resources

All computations are performed using R 4.2.0 on MacOS 12.5 and Python 3.9.7 on Ubuntu 20.04 LTS.

### Institutional review board statement

This study was approved by the Institutional Review Board of the Helsinki University Hospital (journal number 135/13/03/03/2013) and conducted according to the guidelines of the Declaration of Helsinki.

### Informed consent

Participant informed consent was waived because this was a retrospective study. The Institutional Review Board of the Helsinki University Hospital called for an approval by the National Supervisory Authority for Welfare and Health, which was granted (journal number 753/06.01.03.01/2016).

## Results

The initial cohort consists of 842 patients diagnosed with unselected endometrial carcinoma. Following the missing value imputation, excluding subjects that died due to other causes (n = 103) and those that belong to the POLE group (n = 39), where no one died, the final analysis cohort consists of 700 patients. Among 700 patients in the final cohort, 305 (43.6%) belong to the MMRd subgroup, 308 (44%) belong to the NSMP subgroup and 87 (12.4%) belong to the p53ab subgroup. Majority of the tumors are histopathological grade 1–2 (74%) and FIGO stage I disease (73%). The median follow-up time for censored cases is 92 (interquartile range, 78–122) months. There are 182 subjects who had disease recurrence and 147 that died during the follow-up time. Patient demographics are shown in Table 1.

**Table 1:** Patient demographics (n = 700). Feature set I consists of 7 features (FSI), and feature set II consists of 11 features (FSII). FIGO stage refers to the International Federation of Gynecology and Obstetrics staging system, ProMisE stands for Proactive Molecular Risk Classifier for Endometrial Cancer, MMRd – mismatch repair deficient, NSMP – no specific molecular profile, p53ab – p53 aberrant, ER – estrogen receptor status, CD171 – L1 cell adhesion molecule expression status (L1CAM).

Event		No	Yes
N		553	147
**Feature set I (7 features)**			
Age (median [IQR])		66.00 [59.00, 72.00]	71.00 [63.00, 78.00]
FIGO stage (%)	I	455 (82.3)	56 (38.1)
	II	39 (7.1)	11 (7.5)
	III	54 (9.8)	56 (38.1)
	IV	5 (0.9)	24 (16.3)
Histological subgroup (%)	G1-2	463 (83.7)	65 (44.2)
	G3	51 (9.2)	39 (26.5)
	Non-endometrioid	39 (7.1)	43 (29.3)
ProMisE group (%)	MMRd	233 (42.1)	72 (49.0)
	NSMP	274 (49.5)	34 (23.1)
	p53ab	46 (8.3)	41 (27.9)
Deep myometrial invasion (%)	No	383 (69.3)	46 (31.3)
	Yes	170 (30.7)	101 (68.7)
Lymphovascular invasion (%)	No	449 (81.2)	66 (44.9)
	Yes	104 (18.8)	81 (55.1)
Tumor diameter > 3 cm (%)	No	261 (47.2)	22 (15.0)
	Yes	292 (52.8)	125 (85.0)
**Feature set II (Feature set I with 4 additional features)**			
Tumor diameter > 5 cm (%)	No	469 (84.8)	77 (52.4)
	Yes	84 (15.2)	70 (47.6)
Peritoneal washing (%)	Negative	541 (97.8)	113 (76.9)
	Positive	12 (2.2)	34 (23.1)
ER (%)	Negative	52 (9.4)	49 (33.3)
	Positive	501 (90.6)	98 (66.7)
CD171 (%)	Negative	507 (91.7)	99 (67.3)
	Positive	46 (8.3)	48 (32.7)

The multivariable CPH models are compared with the OST models in prediction of the disease-specific survival using two feature sets in four patient cohorts. Variable selection for both feature sets is performed to optimize the CPH discrimination performance. Subsequently, the OST models are fit on the selected feature sets. The feature set I (FSI) consists of seven variables: age, FIGO stage, histological subgroup, ProMisE, deep myometrial invasion, lymphovascular invasion, and tumor diameter > 3 cm. The feature set II (FSII) adds four more variables to the FSI, namely tumor diameter > 5 cm, peritoneal washing status, ER status and CD171 expression (L1CAM, postoperative L1 cell-adhesion molecule expression status).

### Model discrimination

The C-index scores of the CPH and OST models with the corresponding 95% confidence intervals are shown in Table 2.

**Table 2:** C-index of the Cox proportional hazards (CPH) models vs optimal survival tree (OST) models using. Two feature sets are: FSI (7 features) and FSII (11 features). Models **in bold** perform the best in their corresponding cohorts. NSMP refers to no specific molecular profile subtype, MMRd – mismatch repair deficient, p53ab – p53 aberrant. 95% confidence intervals (CI95) are calculated using 1,000 iterations of the ordinary bootstrap with replacement.

Feature Set	Model	Cohort	C-index	CI95
I	CPH	All	0.8425	0.0653
II			0.8489	0.0637
I	OST		0.8493	0.0564
**II**			**0.8586**	**0.0607**
**I**	CPH	NSMP	**0.8376**	**0.1727**
II			0.8325	0.1772
I	OST		0.8368	0.1468
II			0.8284	0.1542
I	CPH	MMRd	0.8200	0.0874
II			0.8251	0.0877
I	OST		0.7886	0.0865
**II**			**0.8843**	**0.0707**
I	CPH	p53ab	0.7636	0.1508
II			0.7744	0.1541
I	OST		0.7246	0.1470
**II**			**0.7936**	**0.1245**

Model discrimination performance is improved by the inclusion of four additional biomarker variables, as indicated by higher C-index scores in the FSII versus FSI feature sets. The OST models trained on the FSII report the highest overall C-index in all ProMisE subcohorts, but the NSMP. Where the CPH model trained on the FSI has the best C-index of 0.8376, followed by the OST model with the C-index of 0.8368. We note that the CPH models trained on the FSI feature set report on average 2.2% higher C-index than the OST models. This trend is reversed in the FSII, where the OST models report on average 2.5% better C-index than the CPH models. The largest C-index increase in the OST models is 10.8% in the MMRd and 8.7% in the p53ab subcohorts, while in the CPH models it is 1.4% in the p53ab subcohort. Overall, non-linear optimal survival tree models benefit more from the additional biomarker data than the linear Cox proportional hazards models.

### Model calibration

**Figure 2:**
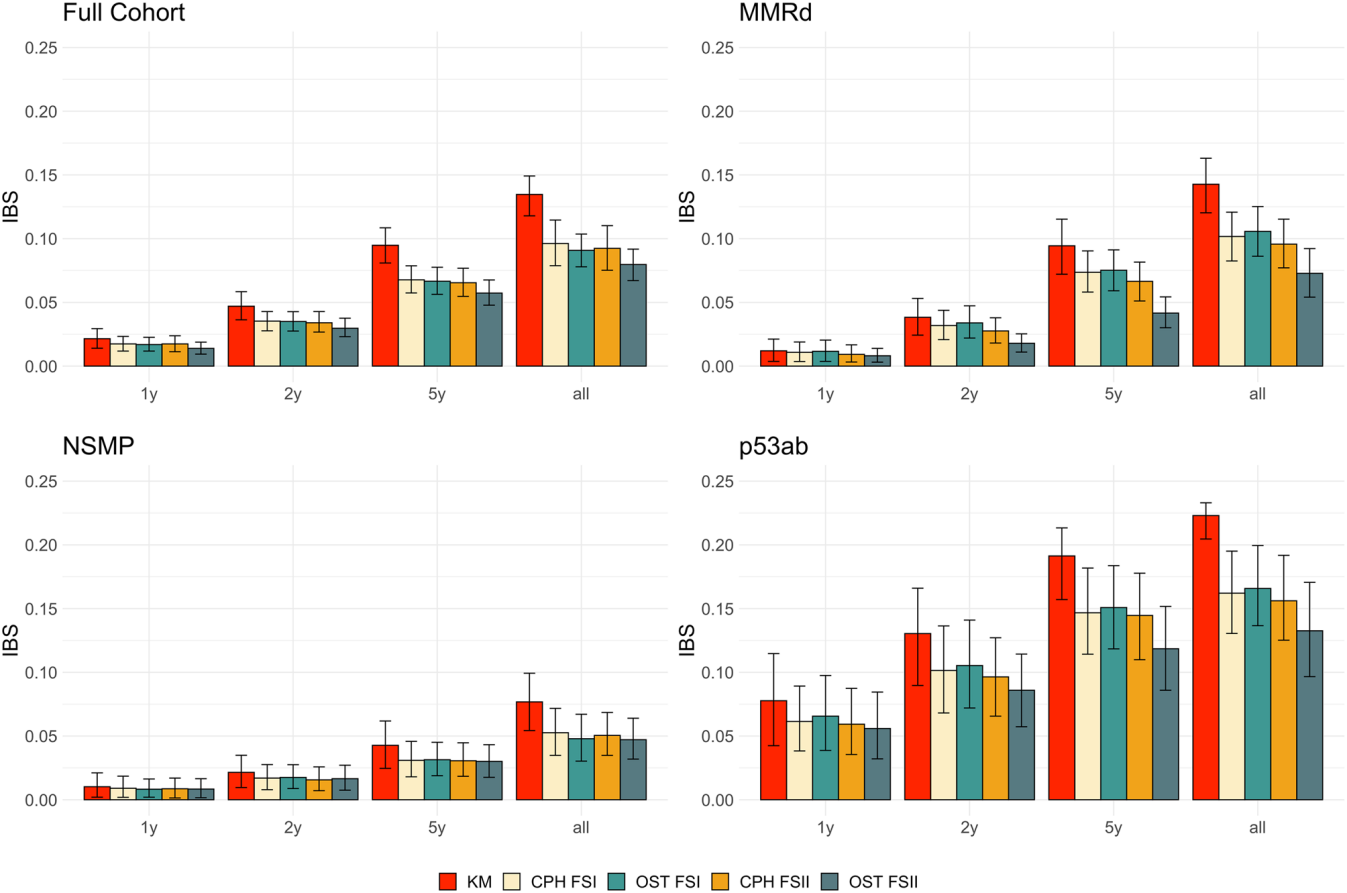
Integrated Brier score (IBS) at 1 year, 2 years, 5 years and 136 months (all) for models trained on four patient cohorts, namely the full cohort, MMRd (mismatch repair deficient), NSMP (no specific molecular profile) and p53ab (p53 aberrant). KM is a non-parametric Kaplan–Meier estimator that may be used as a reference for the parametric models. Cox proportional hazards (CPH) and optimal survival tree (OST) models are trained on two feature sets: FSI with 7 features and FSII with 11 features. Error bars indicate 95% confidence intervals calculated using ordinary bootstrap with replacement, repeated 1000 times.

We report the IBS scores with the 95% confidence intervals for both the CPH and the OST models at 12, 24, and 60 months, and the overall IBS at 136 months of follow-up in Fig. 2 and Supplementary Table 1.

All models across all cohorts and feature sets show better (i.e. lower) IBS at shorter follow-up times, e.g. the IBS scores at 12 months are up to an order of magnitude lower than at 136 months of follow-up. Both OST and CPH model types generally report better IBS scores when trained on a larger feature set (FSII), as compared with the models trained on FSI. The OST models trained on the FSII report 15.4% better IBS at 1 year, 21.6% better IBS at 2 years, 21.0% better IBS at 5 years and 16.2% better IBS at the complete follow-up, as compared with the FSI-trained OST models. The IBS improvements for the CPH models trained on the FSII are 5.7% at 1 year, 7.5% at 2 years, 3.9% at 5 years and 4.4% at the complete follow-up, as compared with the FSI-trained models. Both model types are on par with each other on the FSI feature set, however, the OST models have better IBS scores than the CPH models on the FSII set. The OST models improve more from the additional biomarker data than the CPH models.

### Model interpretation

The hazard ratios with the corresponding 95% confidence intervals of the CPH models trained on a full cohort on two feature sets are in Table 3. It is important to note that the interpretation of the CPH model coefficients should be performed when the proportional hazards (PH) assumption is satisfied. We found evidence that according to the Schoenfeld residual test “non-endometrioid” and “estrogen receptor positive” terms do not satisfy the PH assumption in the CPH models built on the FSI and FSII feature sets. Upon the visual inspection, the violations are minor for both. Further, since both model types pass the global PH test with *p* values of 0.245 and 0.25, respectively, we deem it appropriate to ignore the PH violations.

**Table 3:** Hazard ratios (HR) of the Cox proportional hazards model trained on the full cohort using 7 features (FSI) and 11 features (FSII) with Wald 95% confidence intervals and log-rank test *p* values. FIGO stage refers to the International Federation of Gynecology and Obstetrics staging system, ProMisE stands for Proactive Molecular Risk Classifier for Endometrial Cancer, MMRd – mismatch repair deficient, NSMP – no specific molecular profile, p53ab – p53 aberrant, ER – estrogen receptor status, and CD171 – L1 cell adhesion molecule (L1CAM).

Term	HR on FSI	*p* value	HR on FSII	*p* value
Age	1.04 (1.02–1.06)	1.78E−05	1.04 (1.02–1.06)	9.73E−06
FIGO stage II	1.33 (0.68–2.58)	4.08E−01	1.33 (0.68–2.61)	4.07E−01
FIGO stage III	2.73 (1.78–4.19)	4.06E−06	2.2 (1.4–3.47)	6.80E−04
FIGO stage IV	7.85 (4.35–14.16)	7.52E−12	3.81 (1.9–7.63)	1.64E−04
ProMisE MMRd	1.61 (1.05–2.47)	3.07E−02	1.8 (1.17–2.77)	7.77E−03
ProMisE p53ab	2 (1.2–3.32)	7.57E−03	1.88 (1.13–3.14)	1.51E−02
Histological subgroup G3	2.04 (1.32–3.16)	1.29E−03	1.66 (1.05–2.63)	2.99E−02
Histological subgroup Non-endometrioid	1.35 (0.82–2.21)	2.40E−01	0.95 (0.54–1.68)	8.70E−01
Deep myometrial invasion Yes	1.25 (0.831.88)	2.89E−01	1.13 (0.74–1.73)	5.69E−01
Lymphovascular invasion Yes	1.94 (1.35–2.79)	3.45E−04	2.05 (1.42–2.97)	1.25E−04
Tumor diameter > 3 cm Yes	2.35 (1.44–3.83)	6.49E−04	2.35 (1.37–3.88)	1.39E−03
Tumor diameter > 5 cm Yes			1.29 (0.88–1.9)	1.89E−01
Peritoneal washing positive			2.73 (1.68–4.43)	4.95E−05
ER positive			0.7 (0.45–1.09)	1.14E−01
CD171 positive			1.37 (0.97–2.17)	1.78E−01

Age, more advanced disease stages, larger tumor sizes, deep myometrial invasion, lymphovascular space invasion, positive peritoneal washing, negative ER status and positive CD171 are associated with poor survival^46,47^. The MMRd and p53ab classes are identified as more aggressive EC forms than the NSMP class, with the HR of 1.61 and 2 (1.8 and 1.88 in the FSII), respectively. Similarly, histological subgroup G3 is associated with a higher risk of death than the G1-G2 subgroup with the HR of 2.04 on the FSI and 1.66 on the FSII. Interestingly, the non-endometrioid EC subgroup is not robustly associated with a higher risk in either FSI or FSII feature sets, with HR of 1.35, *p* value 0.24 and HR of 0.95, *p* value 0.87, respectively. This ambiguity in assessing the survival differences between type I and type II tumors has been previously reported in the literature^48,49^.

**Figure 3:**
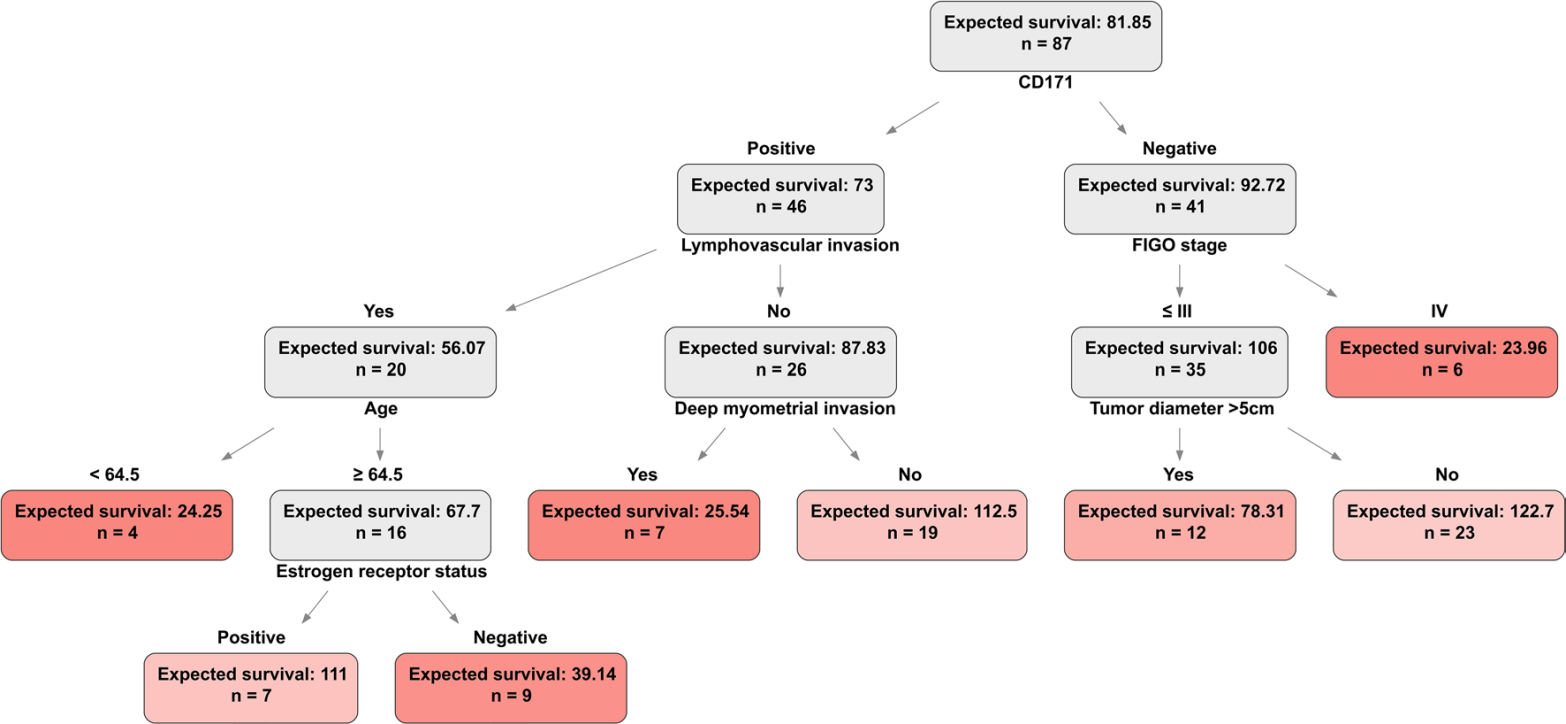
Optimal survival tree for the p53ab subcohort (n = 87) trained on the FSII set consisting of 11 features. Colors indicate leaf (terminal) nodes. Darker hues denote shorter expected survival measured in months and calculated via the integral of a survival function. CD171 refers to the L1 cell adhesion molecule (L1CAM) expression status, FIGO stage is a shorthand for the International Federation of Gynecology and Obstetrics staging system, ER – estrogen receptor status.

We next explore how the tree models may supplement conventional linear methods in the interpretation of EC risk factors by studying the OST and CPH model types trained on the p53ab subcohort and the FSII feature set. We focus on the p53ab subgroup (n = 87), as it shows the largest relative improvement in the C-index from the additional biomarker data in the CPH models (0.7636 vs 0.7744) and the second largest in the OST models (0.7246 vs 0.7936). The CPH IBS values improve by 3% in FSII, whereas for the OST model the improvement is 19%. The HR scores with the 95% confidence intervals of the FSII-trained CPH model are in Table 4. The decision tree for the FSII-trained OST model is in Fig. 3.

**Table 4:** Hazard ratios (HR) of the p53ab subcohort Cox proportional hazards model trained on the FSII with Wald 95% confidence intervals and log-rank test *p* values. FIGO stage refers to the International Federation of Gynecology and Obstetrics staging system, ProMisE stands for Proactive Molecular Risk Classifier for Endometrial Cancer, MMRd – mismatch repair deficient, NSMP – no specific molecular profile, p53ab – p53 aberrant, ER – estrogen receptor status, and CD171 – L1 cell adhesion molecule expression status (L1CAM).

Term	HR on FSII	*p* value
Age	1.02 (0.98–1.07)	3.10E−01
FIGO stage II	106.52 (0-Inf)	1.00E+00
FIGO stage III	6417.32 (0-Inf)	1.00E+00
FIGO stage IV	0 (0-Inf)	1.00E+00
Histological subgroup G3	1.08 (0.35–3.36)	8.90E−01
Histological subgroup Non-endometrioid	1.25 (0.46–3.38)	6.70E−01
Deep myometrial invasion Yes	1.28 (0.55–2.98)	5.60E−01
Lymphovascular invasion Yes	1.19 (0.57–2.52)	6.40E−01
Tumor diameter > 3 cm Yes	2.74 (0.88–8.51)	8.00E−02
Tumor diameter > 5 cm Yes	1.13 (0.52–2.46)	7.60E−01
Peritoneal washing positive	1.94 (0.82–4.58)	1.30E−01
ER positive	1.22 (0.56–2.65)	6.20E−01
CD171 positive	1.12 (0.55–2.3)	7.50E−01

The p53ab CPH model reports a relatively high C-index of 0.7744, but the model coefficients are not always informative and require additional validation. For instance, HR 95% confidence intervals of the “FIGO stage” terms do not have an upper bound and all the coefficients’ *p* values are above the 0.05 threshold of statistical significance (Table 4). Therefore, it is not advised to use this model as is for the downstream tasks that require model interpretation, such as designing nomograms to estimate event probabilities in a clinical setting. The p53ab OST model reports a C-index of 0.7936, and the decision tree path recapitulates some of the existing clinical knowledge (Fig. 3). The OST model selects the CD171 status (L1CAM, L1 cell adhesion molecule expression) as the most informative variable to stratify the cohort on and marks the estrogen receptor status as an important risk factor in the p53ab group. Significance of the ER and CD171 biomarkers in the non-endometrioid p53 aberrant tumors, which are overrepresented in the p53ab ProMisE subcohort with 49.5% of subjects belonging to the non-endometrioid EC subtype versus 11.7% in the full cohort, has been previously reported^47,50,51^. This analysis demonstrates how tree-based ML can supplement and even supersede conventional Cox regression in the EC risk assessment if routinely collected clinical data can be enriched with biomarker information.

## Discussion

We have trained the CPH and OST models on the full, MMRd, NSMP and p53ab ProMisE subcohorts using clinical and extended feature sets. Linear CPH and non-linear OST models trained on seven clinical variables report comparable discrimination performance with the C-index of 0.8425 vs 0.8493 in the complete cohort, and 0.8376 vs 0.8368 in the NSMP subcohort. Model calibration scores are also similar with a 5-year IBS of 0.0677 vs 0.0666 in the full cohort and 0.0309 vs 0.0314 in the NSMP subcohort. In contrast, the CPH models have a better discrimination performance than the OST models with the C-index of 0.82 vs 0.7886 in the MMRd subcohort, and 0.7636 vs 0.7246 in the p53ab subcohort. The CPH models are as well-calibrated as the OST models in these subcohorts with the 5-year IBS of 0.0736 vs 0.0752 and 0.1467 vs 0.1508, respectively. Considering comparable calibration and better discrimination performance, we recommend the Cox proportional hazards regression over the optimal survival tree models for prognostic EC modelling using patient clinical data.

By enriching the clinical variables with biomarker information, namely estrogen receptor and L1 cell adhesion molecule expression status indicators, peritoneal washing status and tumor size < 5 cm, we improve the discrimination and calibration performance of the CPH and OST models. The OST models better utilize additional features and are overall the best EC risk assessment models in the complete (C-index of 0.8586, IBS at 5 years of 0.0573), p53ab (C-index of 0.7936, IBS at 5 years of 0.1185) and MMRd subcohorts (C-index of 0.8843, IBS at 5 years of 0.0416). Further, we show how interpretable OST decision trees may offer insights into the molecular mechanisms of the EC, where the conventional CPH analysis falls short. The p53ab OST model trained on the extended feature set prioritize the L1 cell adhesion molecule and estrogen receptor status indicators as important predictors in the non-endometrioid p53 aberrant tumors. While the p53ab CPH model reports infinitely wide 95% confidence intervals for the FIGO stages and no model coefficients have *p* values below the 0.05 threshold of statistical significance. Therefore, due to overall good discrimination and calibration performance, as well as the model interpretability through the decision path analysis, we recommend the OST method over the CPH regression in the endometrial cancer risk assessment, if patient clinical profiles can be enriched with biomarker data.

There are several limitations in our study. Firstly, better prognostic survival models could be created if we had access to an external validation cohort^52,53^. In general, we hope that the research community could share anonymized patient datasets more freely, as open-access initiatives contribute to the development of better prognostic prediction models^54^. Further, in addition to the IBS, we are interested in exploring other model calibration measures, such as the integrated calibration index or standardized mortality ratio^55^. The third limitation stems from the methodological difficulties in the assessment of data imputation methods and their downstream effects. In this work we did not perform any formal tests to identify the missingness type, assuming missing at random for all explanatory covariates^56^. We performed an ad hoc assessment of imputation quality by comparing imputed variable distributions with those in the complete case cohorts. More robust and comprehensive methods for the assessment of data imputation techniques are needed^57^.

## Conclusion

We show that the Cox proportional hazards and optimal survival tree models are well-suited for the prognostic survival modeling of endometrial carcinoma. The Cox proportional hazards regression is the method of choice for the EC risk assessment on the clinical feature set, consisting of seven variables. Extending clinical variables with the ER and L1CAM status indicators, tumor diameter > 5 cm and peritoneal washing status, improves the discrimination and calibration performance in both model types. Due to the overall best C-index and IBS scores, as well as visually interpretable structure, we recommend optimal survival tree models if clinical variable set can be supplemented with additional biomarker data. Finally, we stress the importance of reporting model discrimination and calibration metrics to promote further adoption of ML prognostic models into the clinical practice.

## Supplementary Information

Supplementary Information 1.

Supplementary Information 2.

Supplementary Information 3.

## Data Availability

The code and individual survival probabilities estimated using the OST and CPH models are available on https://github.com/netphar/survival_analysis. The datasets used and/or analyzed during the current study available from the corresponding author on reasonable request.
