# Supplementary material for: Interpretable prognostic modeling of endometrial cancer
Source: Sci Rep. 2022 Dec 13;12:21543. doi: 10.1038/s41598-022-26134-w (PMC9747711; doi:10.1038/s41598-022-26134-w)
Supplement: Supplementary file 1 — Supplementary Information 1. [file 41598_2022_26134_MOESM1_ESM.docx]

## Supplementary Materials

### Extended variable information

Each patient is described with a feature vector consisting of 43 variables, out of which 33 are categorical and 10 are numeric. Numeric variables are:

- Age;
- β-subunit of human chorionic gonadotropin (βhCG);
- Body mass index (BMI);
- Cancer antigen 125 (CA-125);
- Creatinine;
- Hemoglobin;
- Hematocrit;
- Human chorionic gonadotropin (hCG);
- Leukocytes;
- Thrombocytes.

Categorical variables are binary, unless stated otherwise:

- Adjuvant therapy (chemotherapy, vaginal brachytherapy, whole pelvic radiotherapy, whole pelvic radiotherapy with chemotherapy);
- ARID1a protein loss;
- CTNNB1 nuclear expression (negative, diffuse, focal);
- HER-2/neu expression (negative, diffuse and strong, diffuse and weak, focal);
- Histological subgroup (grade 1-2, grade 3, non-endometrioid);
- HNF1b positivity;
- Presence of endometrial hyperplasia;
- E-cadherin expression (negative, normal, weak);
- Estrogen (ER) and progesterone receptor (PR) expression;
- FIGO stage (I-II-III-IV);
- Final histology (carcinosarcoma, clear cell carcinoma, endometrioid carcinoma, serous carcinoma, undifferentiated carcinoma);
- Iliacal lymphadenectomy;
- KRAS mutation;
- Lymphadenectomy;
- p16 expression (negative, diffuse, focal);
- Paraaortic lymp node status;
- Peritoneal washing status;
- Postoperative Mayo criterion;
- Preoperative histology (low and high risk);
- Pre- and postoperative L1CAM (CD171) expression;
- ProMisE class (MMRd, NSMP, p53ab, POLE);
- Smoking status;
- Tumor infiltrating leukocytes (none, moderate, abundant);
- Tumor infiltrating leukocytes PD-L1 expression binarized at 1% and 10%;
- Uterine risk factors:
  - Tumor diameter at 2cm/3cm/5cm levels;
  - Deep myometrial invasion (≥50%);
  - Lymphovascular space invasion;
  - Myometrial invasion with levels <33%, 33%-66%, >66%;
- Vimentin expression (negative, diffuse, focal);

More detailed information regarding the study cohort is available in prior work [58,59].

### IBS model scores

Supplementary Table 1. Integrated Brier score (IBS) of Cox proportional hazards (CPH) and optimal survival tree (OST) models at 1, 2, 5 years and at the complete follow-up. The models are trained on 7 features (FSI) and 11 features (FSII). MMRd – mismatch repair deficient, NSMP – no specific molecular profile, p53ab – p53 aberrant.

|  | **Model** | **Cohort** | **IBS FSI** | **IBS FSII** |
| --- | --- | --- | --- | --- |
| IBS at 1 year | CPH | all | 0.0174 | 0.0174 |
| IBS at 2 years |  |  | 0.0353 | 0.034 |
| IBS at 5 years |  |  | 0.0677 | 0.0654 |
| overall IBS |  |  | 0.0962 | 0.0924 |
| IBS at 1 year | OST |  | 0.0169 | 0.0139 |
| IBS at 2 years |  |  | 0.035 | 0.0297 |
| IBS at 5 years |  |  | 0.0666 | 0.0573 |
| overall IBS |  |  | 0.0908 | 0.0797 |
| IBS at 1 year | CPH | MMRd | 0.0108 | 0.0092 |
| IBS at 2 years |  |  | 0.0318 | 0.0276 |
| IBS at 5 years |  |  | 0.0736 | 0.0665 |
| overall IBS |  |  | 0.1017 | 0.0957 |
| IBS at 1 year | OST |  | 0.0116 | 0.0081 |
| IBS at 2 years |  |  | 0.0339 | 0.0179 |
| IBS at 5 years |  |  | 0.0752 | 0.0416 |
| overall IBS |  |  | 0.1057 | 0.0728 |
| IBS at 1 year | CPH | NSMP | 0.009 | 0.0086 |
| IBS at 2 years |  |  | 0.017 | 0.0156 |
| IBS at 5 years |  |  | 0.0309 | 0.0306 |
| overall IBS |  |  | 0.0526 | 0.0505 |
| IBS at 1 year | OST |  | 0.0083 | 0.0084 |
| IBS at 2 years |  |  | 0.0175 | 0.0165 |
| IBS at 5 years |  |  | 0.0314 | 0.0301 |
| overall IBS |  |  | 0.0479 | 0.0471 |
| IBS at 1 year | CPH | p53ab | 0.0614 | 0.0593 |
| IBS at 2 years |  |  | 0.1014 | 0.0964 |
| IBS at 5 years |  |  | 0.1467 | 0.1466 |
| overall IBS |  |  | 0.1621 | 0.1561 |
| IBS at 1 year | OST |  | 0.0656 | 0.0559 |
| IBS at 2 years |  |  | 0.1053 | 0.0859 |
| IBS at 5 years |  |  | 0.1508 | 0.1185 |
| overall IBS |  |  | 0.1658 | 0.1326 |

### Additional ML models

We trained 9 survival analysis models on a full patient cohort. Supplementary Figure 1 displays model performance in the prediction of disease-specific survival in the full EC cohort using C-index as a metric. The cohort is preprocessed as indicated in the Materials and Methods section with three modifications. Firstly, we extended the FSII feature set with “leukocytes”, “hemoglobin”, “thrombocytes” variables, as well as thee CA125 and PR status indicators for a total of 16 variables. Secondly, we normalized the numerical variables to their z-scores, instead of winsorizing them. The z-score is calculated as follows:

$$z_{score}= \frac{x-\mu}{2\times s}$$

- *x* - numerical variable;
- *μ* - sample mean of *x*;
- *s* - sample standard deviation of *x*.

factor 2 is used in the denominator to make sure that the feature values are on the same scale as binary variables (0, 1), such that the distribution of transformed numerical variables has a mean of 0 and variance of 0.25. Thirdly, we use one-hot encoding to transform the categorical variables.

In addition to the CPH and OST models, seven tested models are:

- CoxNet model that extends linear Cox model by introducing L_1_ and L_2_ penalties to adjust for the impact of outliers (Simon et al, 2011);
- Gradient Boosting Machine (GBM) model is an ensemble method that fits shallow decision trees in a stage-wise manner, such that each subsequent tree is fitted on the residuals (errors) from the previous tree (Chen and Guestrin, 2016);
- Three models are based on Support Vector Machines (SVM) that perform survival modeling in the expanded feature space. We use SVMs with a linear kernel (linSVM), radial basis function kernel (rbfSVM) and minimal Lipschitz smoothness strategy linear kernel (lipSVM) (van Belle, 2011);
- Random Survival Forest (RF) (Pölsterl, 2020);
- Stacked model that combines the above mentioned models by averaging their predictions.

We optimize hyperparameters of all ML models using 5-fold cross-validation, and then refit the models on the complete cohorts. The CI intervals are given as +/- 1 standard deviation of the C-index calculated via 100 iterations of ordinary bootstrap with replacement.


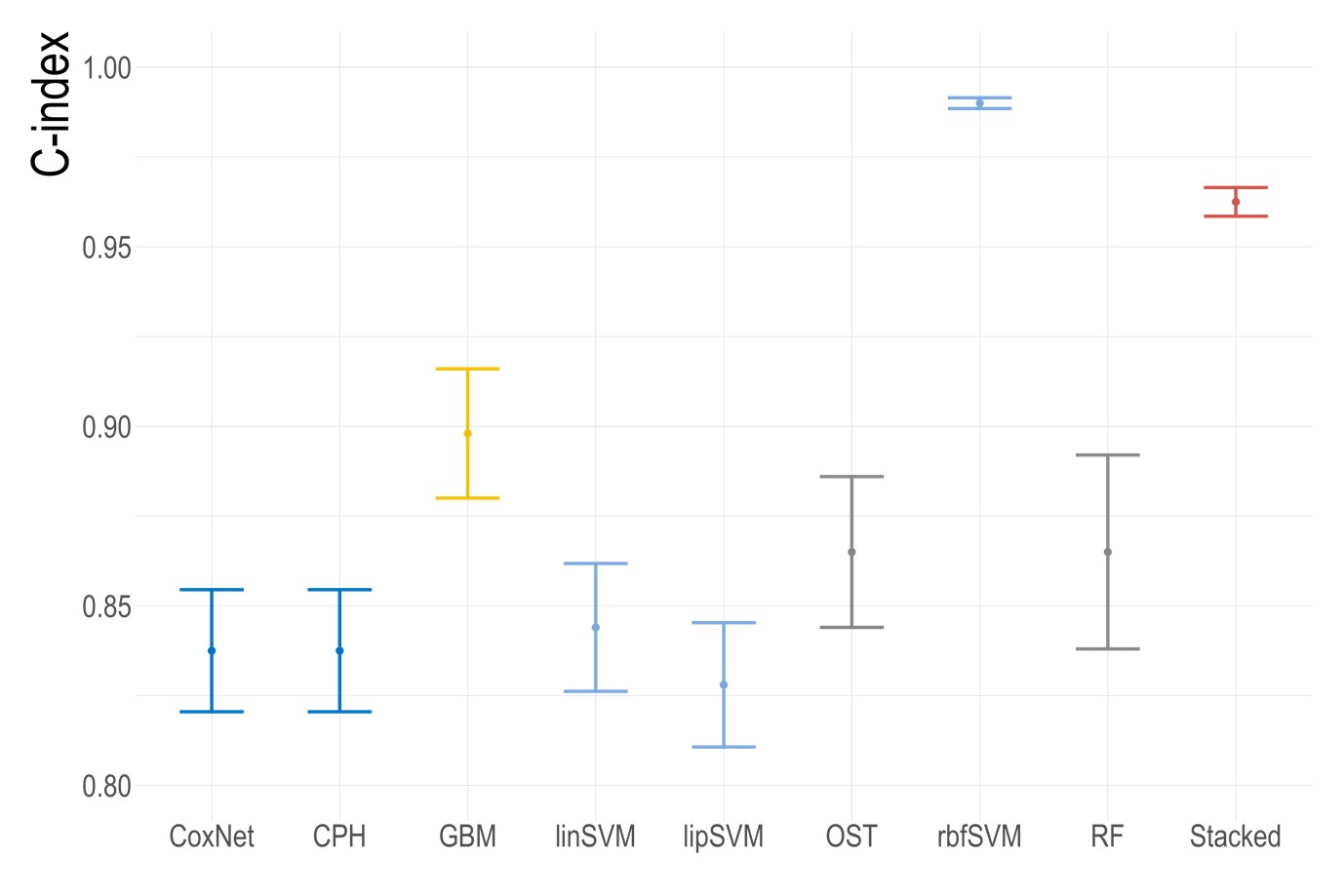


Supplementary Figure 1. C-index of nine prognostic models trained on the full cohort (n = 700). The 95% confidence intervals are given as +/- 1 standard deviation calculated via 100 iterations of ordinary bootstrap with replacement. CPH is Cox proportional hazards regression, linSVM – linear kernel Support Vector Machine, lipSVM - minimal Lipschitz smoothness strategy linear kernel Support Vector Machine, OST – optimal survival tree, rbfSVM – radial basis kernel Support Vector Machine, RF – random forest.

As seen in Supplementary Figure 1, rbfSVM leads to almost perfect C-index scores. Further, the GBM and Stacked models significantly outperform the CPH and OST models, which are the two main methods used in our work. Despite such impressive performance scores of the SVM, GBM and Stacked models, these methods are not easily interpretable, as such, they do not circumvent the main (from our point of view) limitation of ML methods in prognostic survival modeling - their “black box” nature. Difficulties in the model in interpretability are further compounded by insufficient performance reporting in the literature. These factors severely limit a more widespread use of ML and DL models in the clinical practice [18].
