# Supplementary figures and images for: Interpretable prognostic modeling of endometrial cancer
Source: Sci Rep. 2022 Dec 13;12:21543. doi: 10.1038/s41598-022-26134-w (PMC9747711; doi:10.1038/s41598-022-26134-w)

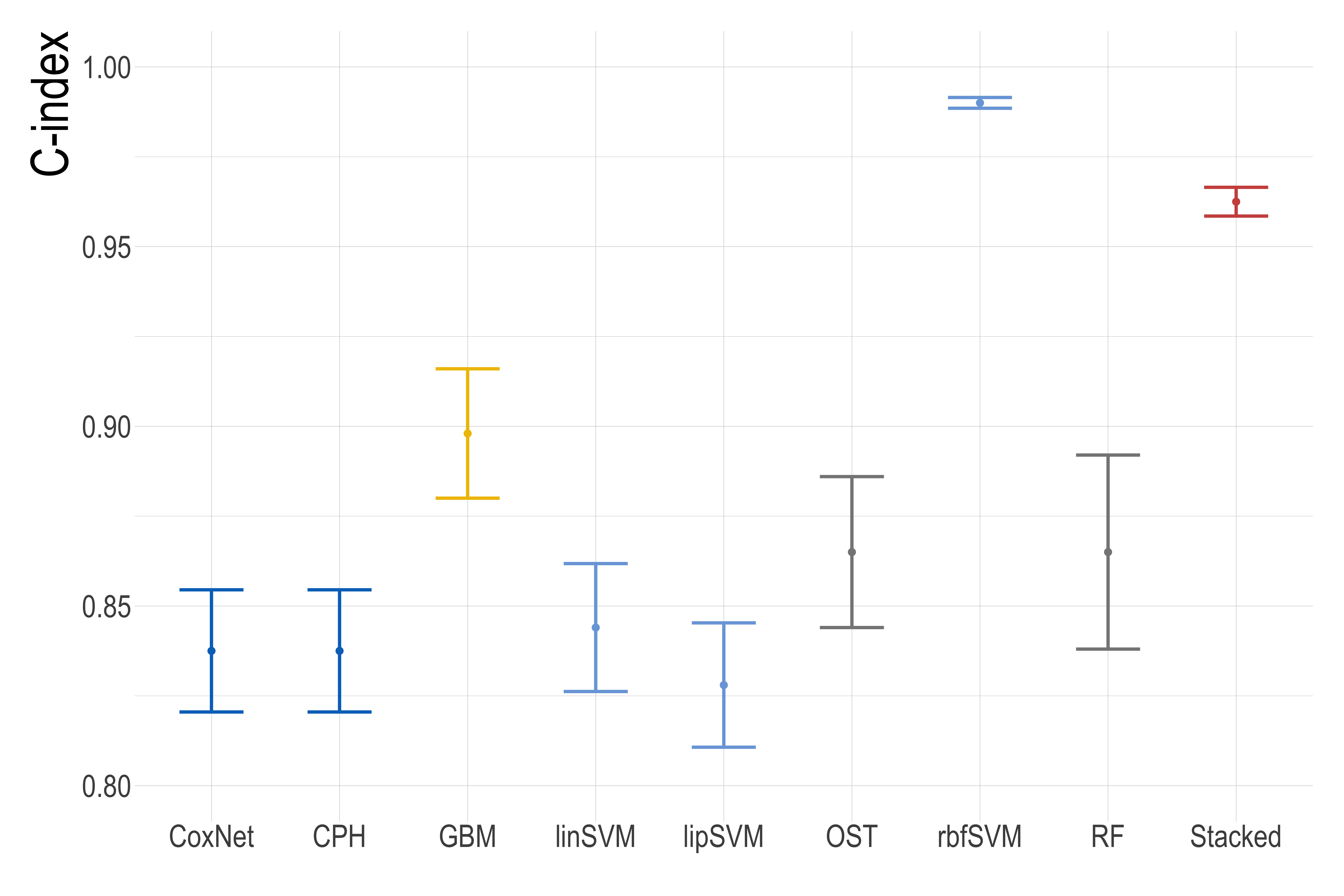

Supplement: Supplementary file 2 — Supplementary Information 2. [file 41598_2022_26134_MOESM2_ESM.png]
